# Light‐Driven Cascade Mitochondria‐to‐Nucleus Photosensitization in Cancer Cell Ablation

**DOI:** 10.1002/advs.202004379

**Published:** 2021-02-08

**Authors:** Kang‐Nan Wang, Liu‐Yi Liu, Guobin Qi, Xi‐Juan Chao, Wen Ma, Zhiqiang Yu, Qiling Pan, Zong‐Wan Mao, Bin Liu

**Affiliations:** ^1^ Shunde Hospital, Southern Medical University (The First People's Hospital of Shunde) Foshan Guangdong 528308 China; ^2^ Department of Chemical and Biomolecular Engineering National University of Singapore 4 Engineering Drive 4 Singapore 117585 Singapore; ^3^ MOE Key Laboratory of Bioinorganic and Synthetic Chemistry School of Chemistry Sun Yat‐sen University Guangzhou 510275 China; ^4^ Guangdong Provincial Key Laboratory of New Drug Screening School of Pharmaceutical Sciences Southern Medical University Guangzhou 510515 China; ^5^ Joint School of National University of Singapore and Tianjin University International Campus of Tianjin University Binhai New City Fuzhou 350207 China

**Keywords:** functional iridium complex, mitochondria‐to‐nucleus translocation, nucleic acid damage, photodynamic therapy

## Abstract

Nuclei and mitochondria are the only cellular organelles containing genes, which are specific targets for efficient cancer therapy. So far, several photosensitizers have been reported for mitochondria targeting, and another few have been reported for nuclei targeting. However, none have been reported for photosensitization in both mitochondria and nucleus, especially in cascade mode, which can significantly reduce the photosensitizers needed for maximal treatment effect. Herein, a light‐driven, mitochondria‐to‐nucleus cascade dual organelle cancer cell ablation strategy is reported. A functionalized iridium complex, named BT‐Ir, is designed as a photosensitizer, which targets mitochondria first for photosensitization and subsequently is translocated to a cell nucleus for continuous photodynamic cancer cell ablation. This strategy opens new opportunities for efficient photodynamic therapy.

## Graphical abstract

Herein, a functionalized iridium complex, named BT‐Ir, is designed as a light‐driven, mitochondria‐to‐nucleus cascade organelle targeted photosensitizer, which targets mitochondria first for photosensitization and subsequently is translocated to the cell nucleus for continuous photodynamic cancer cell ablation. This strategy opens new opportunities for efficient photodynamic therapy.

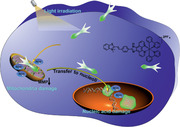

## 1 Introduction

Photodynamic therapy (PDT) involves the production of reactive oxygen species (ROS) by the irradiation of photosensitizers (PSs), which is considered as one of the most momentous strategies to eliminate cancer cells with minimal invasiveness and precise spatiotemporal selectivity.^[^ ^1^, ^2^, ^3^, ^4^ ^]^ The primary cytotoxic agents involved in this photodynamic process are reactive oxygens, such as singlet oxygen (^1^O_2_), hydroxyl radicals (OH^●^), etc.^[^ ^5^, ^6^ ^]^ The incorporation of heavy atoms into the molecular structures is one of the widely used approaches to improve the ROS yield of PSs.^[^ ^7^, ^8^, ^9^, ^10^ ^]^ The PSs with heavy metal elements have played an important role in anti‐tumor and antibacterial aspects.^[^ ^11^, ^12^, ^13^, ^14^, ^15^ ^]^ However, the presence of heavy metal, such as platinum, gold, copper, ruthenium, and iridium, has often been reported to cause increased “dark toxicity”.^[^ ^16^ ^]^ Therefore, strategies that could offer an efficient supply of ROS at low dosage are highly desirable.

It was reported that targeted PDT at the lesions, including tissues, tumor cells, and especially organelles, was able to improve the curative effect of the PDT.^[^ ^17^, ^18^, ^19^, ^20^ ^]^ Therefore, subcellular compartments, including the mitochondria, nucleus, etc., have become targeting sites to enhance anticancer efficiency.^[^ ^17^, ^21^, ^22^, ^23^ ^]^ As the central regulator of cell growth and proliferation, nucleus, especially the nucleoli, is considered to be the most aspirational target for most of the therapeutic drugs, especially for malignant tumors.^[^ ^24^, ^25^, ^26^ ^]^ For instance, nucleus‐targeting anticancer drugs, such as doxorubicin,^[^ ^27^ ^]^ paclitaxel,^[^ ^28^ ^]^ carboplatin and cisplatin,^[^ ^29^, ^30^ ^]^ etc., induce cell death by interacting with the DNA helix or its related enzymes. Only very few nucleus targeting PSs for cancer therapy have been developed so far.^[^ ^26^, ^31^, ^32^ ^]^ As a central role in many metabolic tasks, mitochondria also has served as the targeting site for efficient cancer therapy.^[^ ^19^, ^33^, ^34^, ^35^ ^]^ Some PSs inducing mitochondrial damage through ROS also have been developed to combat cancer with good effect.^[^ ^18^, ^36^, ^37^, ^38^, ^39^ ^]^ As both mitochondria and nucleus have been proven as effective targets for cancer treatment, it is interesting if one could develop PSs that can simultaneously target both, which has not been realized so far. An even more exciting strategy is to develop photosensitizers that can target mitochondria and subsequently be translocated into the nucleus so that the same amount of PSs can be used twice to damage genes in both mitochondria and nucleus through photosensitization. This will allow us to use limited amount of PSs for a maximized killing effect, which is also beneficial for the overall biocompatibility of the PSs when a low amount is needed.

**Scheme 1:**
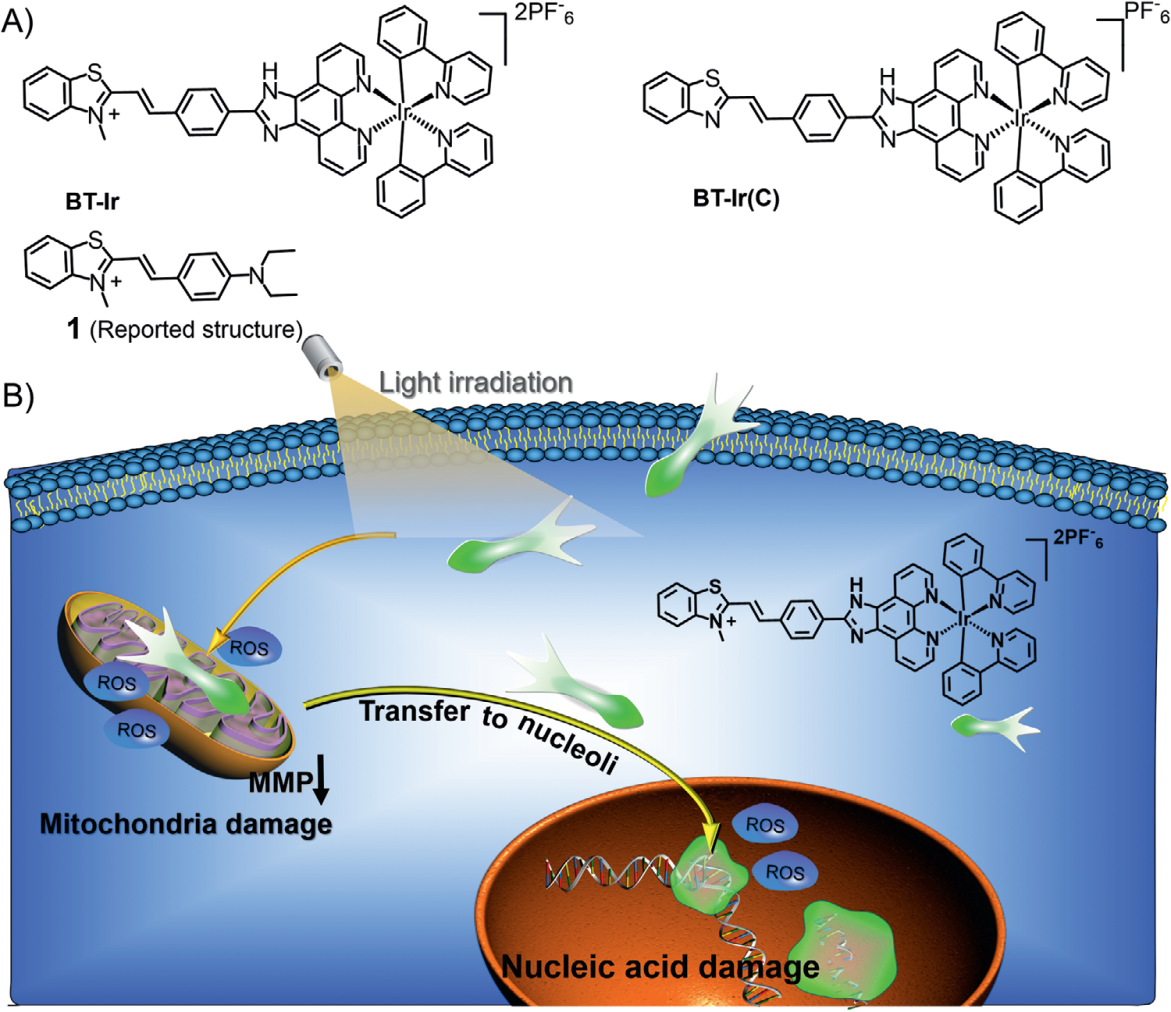
A) Chemical structures of BT‐Ir, BT‐Ir(C), and compound 1. B) Schematic representation of light‐driven cascade mitochondria‐to‐nucleoli photosensitization in cell ablation. Moderate ROS generation upon photoirradiation promotes mitochondria damage, thus resulting in BT‐Ir escape from the mitochondria to the nucleolus, further damaging nucleic acids under light irradiation.

In order to achieve cascade photosensitization in the mitochondria and nucleus, herein, we rationally designed an iridium complex, named BT‐Ir (**Scheme** **1**). The functional BT‐Ir can not only damage mitochondria through ROS under light illumination, but also offers controllable mitochondria‐to‐nucleus translocation to damage nucleic acids, which can effectively ablate cancer cells. An analogue of BT‐Ir, the BT‐Ir(C) (Scheme 1), enriched in mitochondria under darkness and irradiation conditions, showed a much weaker cancer cell killing ability. A detailed study reveals that the controllable nucleus enrichment of BT‐Ir can efficiently induce nucleic acid damage and cell death via apoptosis.

## 2 Results and Discussion

To realize mitochondria‐to‐nucleus translocation‐based controllable cancer cell ablation, the therapeutic molecule should be designed to be highly specific to mitochondria and efficiently bind to nucleic acids. Besides, a specific mechanism is required to change the molecular affinity for mitochondria and nucleus in a controllable manner. It is known that the cationic molecules can interact with mitochondria and other subcellular organelles due to an electrophoresis effect, and can bind with negatively charged nucleic acids via electrostatic interaction.^[^ ^40^, ^41^, ^42^, ^43^ ^]^ By optimizing the molecular structure, adjusting the binding ability between the cationic molecules and phospholipid bilayer membrane, probes can be designed to target different subcellular organelles.^[^ ^44^, ^45^ ^]^ For example, as the organelle membrane potential could change under different physiological/pathological conditions, some cationic molecules could be transferred between different organelles.^[^ ^46^, ^47^, ^48^, ^49^ ^]^ In this regard, the molecules targeting mitochondria can be detached when the mitochondrial membrane potential (MMP) is affected by physiology/pathology, which provides a rare opportunity to realize mitochondria‐to‐nucleoli translocation when the molecule is properly designed.

It is known that the cyclometallic iridium(III) complex can be well enriched on mitochondria through MMP.^[^ ^50^, ^51^, ^52^ ^]^ In addition, we and other groups have confirmed that benzothiazolium derivatives have a good nuclear targeting potential.^[^ ^53^, ^54^, ^55^, ^56^, ^57^ ^]^ By using the benzothiazolium probe 1^[^ ^57^ ^]^ as a nuclear targeting unit, we integrate it with an iridium complex yielded BT‐Ir (Scheme 1). An analogue compound of BT‐Ir(C) with a neutral benzothiazole unit was also synthesized as control. The characterizations of the BT‐Ir and BT‐Ir(C) are described in Scheme S1 and Figures S1–S6, Supporting Information, and the results revealed the right structure and high purity of all compounds. The UV‐vis absorption and emission maxima of the BT‐Ir (C) are at 385 and 465 nm, respectively. Due to the protonation of the benzothiazole unit, the absorption and emission maxima of the BT‐Ir are redshifted to 452 and 710 nm, respectively. The BT‐Ir has a large stokes shift of more than 200 nm, and its broad emission spectrum covers the far and NIR channel, which facilitates the study of the dynamic behavior of BT‐Ir in subcellular organelles (Figures S7 and S8, Supporting Information).

**Figure 1:**
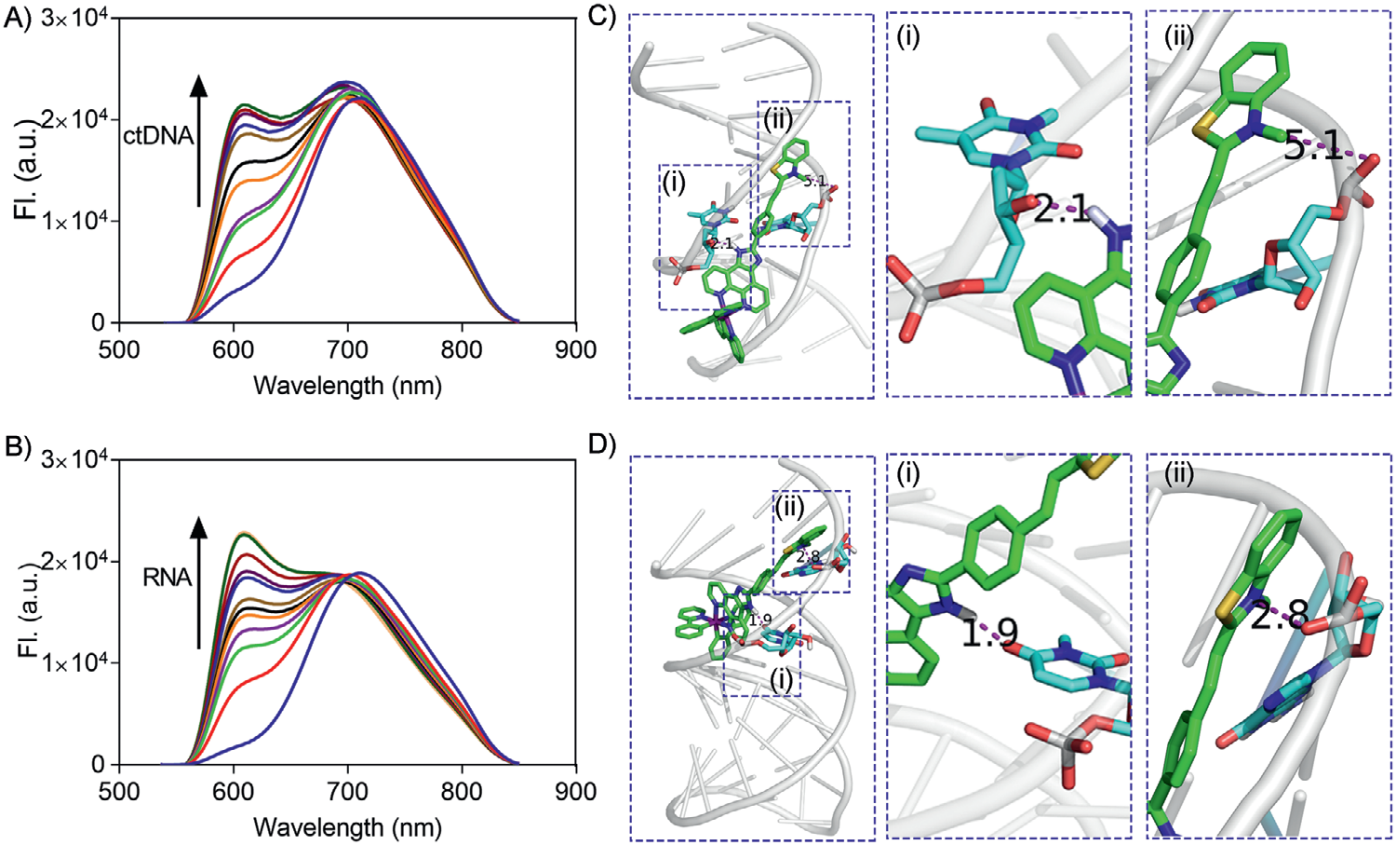
Emission spectra of BT‐Ir titrated by A) ctDNA (40 µg mL^−1^) and B) RNA (48 µg mL^−1^) in 10 mm Tris‐HCl buffer (pH 7.4, 100 mm K^+^) at *λ*_ex_ = 405 nm. The binding modes of C) DNA/BT‐Ir complex and D) RNA/BT‐Ir complex. The molecular modeling calculations based on the optimized structure of BT‐Ir and DNA structure (5ʹ‐CAATCGGATCGAATTCGATCCGATTG‐3ʹ, PDB code: 5ju4) and RNA structure (5ʹ‐GAGUAGAAACAAGGCUUCGGCCUGCUUUUGCU‐3ʹ, PDB code: 2lwk) using AutoDock 4.2 software (See Table S1, Supporting Information, in ESI).

With the addition of DNA or RNA, the fluorescence of the BT‐Ir at ≈620 nm was significantly enhanced; in contrast, a negligible fluorescence change was observed with the addition of bovine serum albumin (BSA), indicating the high affinity of the BT‐Ir to nucleic acids (**Figure** **1**A,B and Figure S9, Supporting Information). However, the control compound BT‐Ir(C) showed inert fluorescence responses to both DNA/RNA and protein (Figure S10, Supporting Information), which indicated that the positively charged benzothiazolium unit could help increase the binding of the complex to nucleic acids. A molecular modeling calculation indicated that the BT‐Ir molecules were incorporated into the hydrophobic minor grooves of DNA or inserted into the major hydrophobic grooves of RNA with electrostatic interaction (the quaternized nitrogen on benzothiazolium unit of BT‐Ir and nucleic acids^[^ ^54^, ^57^ ^]^) and hydrogen bond (the indolyl‐NH on the O‐phenanthroline ligands of BT‐Ir and nucleic acids) (Figure 1C,D and Table S1, Supporting Information). The binding between the BT‐Ir and nucleic acids limits molecular motions of the BT‐Ir, resulting in intensified fluorescence. These results lay the foundation for BT‐Ir to light up nucleic acids in living cells.

**Figure 2:**
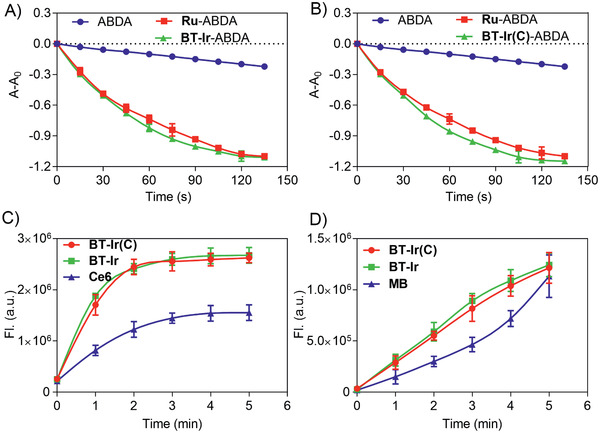
A,B) Rate of decay of ABDA sensitized by control, Ru ([Ru(bpy)_3_]Cl_2_, 10 µm), A) BT‐Ir (10 µm), and B) BT‐Ir(C) (10 µm) in aerated disodium hydrogen phosphate/citric acid buffer solutions, pH = 7.4, where *A*_0_ and *A* are the absorbance of ABDA at 378 nm before and after light irradiation. C) Fluorescence intensity of SOSG (10 µm) at 525 nm as a function of irradiation time in the presence of BT‐Ir, BT‐Ir(C), and the control Ce6 (10 µm). D) Fluorescence intensity of HPF (10 µm) at 516 nm as a function of irradiation time in the presence of BT‐Ir, BT‐Ir(C), and the control MB (10 µm).

The ROS generation properties of the BT‐Ir and BT‐Ir(C) in solution were subsequently investigated using 9,10‐anthracenediyl‐bis(methylene) dimalonic acid (ABDA) (50 µm) as the ROS indicator. As shown in **Figure** **2**A,B, upon light irradiation, the absorption of the ABDA is gradually decreased in the presence of BT‐Ir and BT‐Ir(C) (pH = 7.4), which is similar to that of the commonly used metal photosensitizer [Ru(bpy)_3_]Cl_2_, a standard used for ROS yield of metal complexes.^[^ ^58^ ^]^ Due to the presence of protonation/deprotonation effect of indolyl‐NH on the O‐phenanthroline ligands, the BT‐Ir has a more stable conjugated structure in an acidic environment. As shown in Figure S11, Supporting Information, at the buffer solution with pH 6.8, the absorption of the ABDA in the presence of BT‐Ir decreased significantly within 2 min, indicating highly efficient ROS generation in the acidic environment, which is more conducive to kill cancer cells as the tumor microenvironment is acidic.^[^ ^15^ ^]^ Although ^1^O_2_ (type II) is considered to be the predominant primary ROS involved in PDT, there is growing evidence that other ROS (type I, including superoxide anion (O_2_^−^), hydroxyl radical (HO^●^), hydrogen peroxide (H_2_O_2_), etc.), may also be produced during the PDT process and play important roles in cell death.^[^ ^6^ ^]^ Methylene blue (MB) and Ce6 are therefore selected as the references for type I and type II PSs, respectively. The ^1^O_2_ measurement results of the BT‐Ir and BT‐Ir(C) using singlet oxygen sensor green (SOSG) were consistent with that of the ABDA, and showed more efficient ^1^O_2_ production than that of the control Ce6 (Figure 2C). The OH^●^ production of the BT‐Ir and BT‐Ir(C) measured using hydroxyphenyl fluorescein (HPF) as the indicator is comparable to that of MB (Figure 2D). These results indicate that both iridium complexes are type I and type II PSs, and their effective ROS generation ability is ideal for the cascade damage of mitochondria and nucleus in living cells.

**Figure 3:**
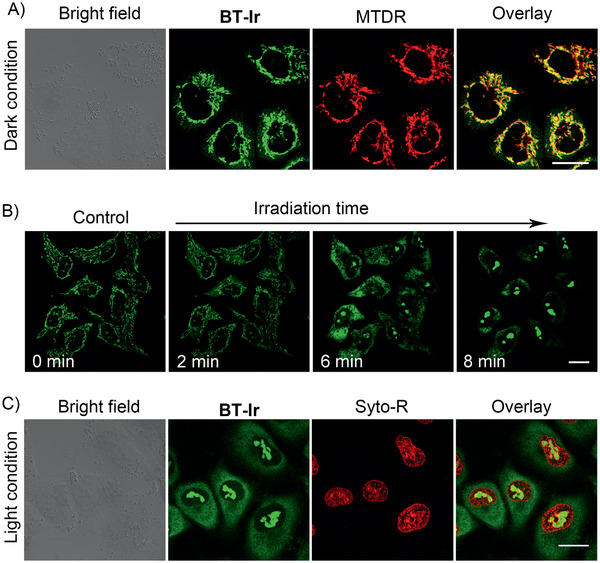
A) Confocal images of A549 cells co‐stained with BT‐Ir and MTDR under a dark condition. B) Confocal images of A549 cells stained with BT‐Ir before (control) and after light irradiation. A549 cells were irradiated at 405 nm (15 mW cm^–2^), irradiated 5 s every 2 min for a total of 10 cycles (Video S1, Supporting Information). C) Confocal imaging of A549 cells co‐stained with BT‐Ir and Syto‐R under 405 nm light irradiation condition. For BT‐Ir, *λ*_ex_: 405 nm, *λ*_em_: 620 ± 20 nm (pseudo green‐color); For MTDR, *λ*_ex_: 633 nm; *λ*_em_: 650 ± 10 nm. For Syto‐R, *λ*_ex_: 633 nm, *λ*_em_: 650 ± 10 nm. Scale bar: 20 µm.

As reported in the literature,^[^ ^57^ ^]^ probe 1 can be perfectly enriched in the nucleolus after incubation for 10 min under dark conditions (Figure S12, Supporting Information). To investigate the location of the BT‐Ir in living cells, A549 cells were used to incubate with the BT‐Ir, and this was followed by co‐staining with Mito Tracker Deep Red (MTDR). As shown in **Figure** **3**A, the green fluorescence from the BT‐Ir overlays well with the red fluorescence from the MTDR with a Pearson's correlation coefficient (PCC) of 0.86, implying that the BT‐Ir was mainly targeting the cell mitochondria. During the co‐localization experiment, an interesting phenomenon was also observed. As shown in Figure 3B and Video S1, Supporting Information, at the initial stage, the fluorescence of the BT‐Ir was completely overlaid with the mitochondrial tracker. However, after exposure to 405 nm irradiation for 5 s, the green fluorescence of mitochondria stained with BT‐Ir was gradually weakened and diffused, and the nucleoli of A549 cells were gradually lightened up. With the extended irradiation time, the BT‐Ir molecules, escaped from the mitochondria, were utterly diffused in the cytoplasm, indicating the dissociation of the BT‐Ir from the mitochondria. To accurately confirm the translocation of the BT‐Ir to the nucleoli, A549 cells were co‐stained with Syto59 Red (Syto‐R, a membrane‐permeable deep red fluorescent nucleic acid probe) after a 20 min light irradiation. As shown in Figure 3C, the red fluorescence from the Syto‐R in the nucleolus is completely overlaid with the BT‐Ir. All these results highlight that the BT‐Ir can escape from the mitochondria and enter the nuclei under light irradiation. In contrast, the fluorescence of the mitochondria located with the BT‐Ir(C) does not transfer to the nuclei before or after irradiation (Figure S13, Supporting Information).

**Figure 4:**
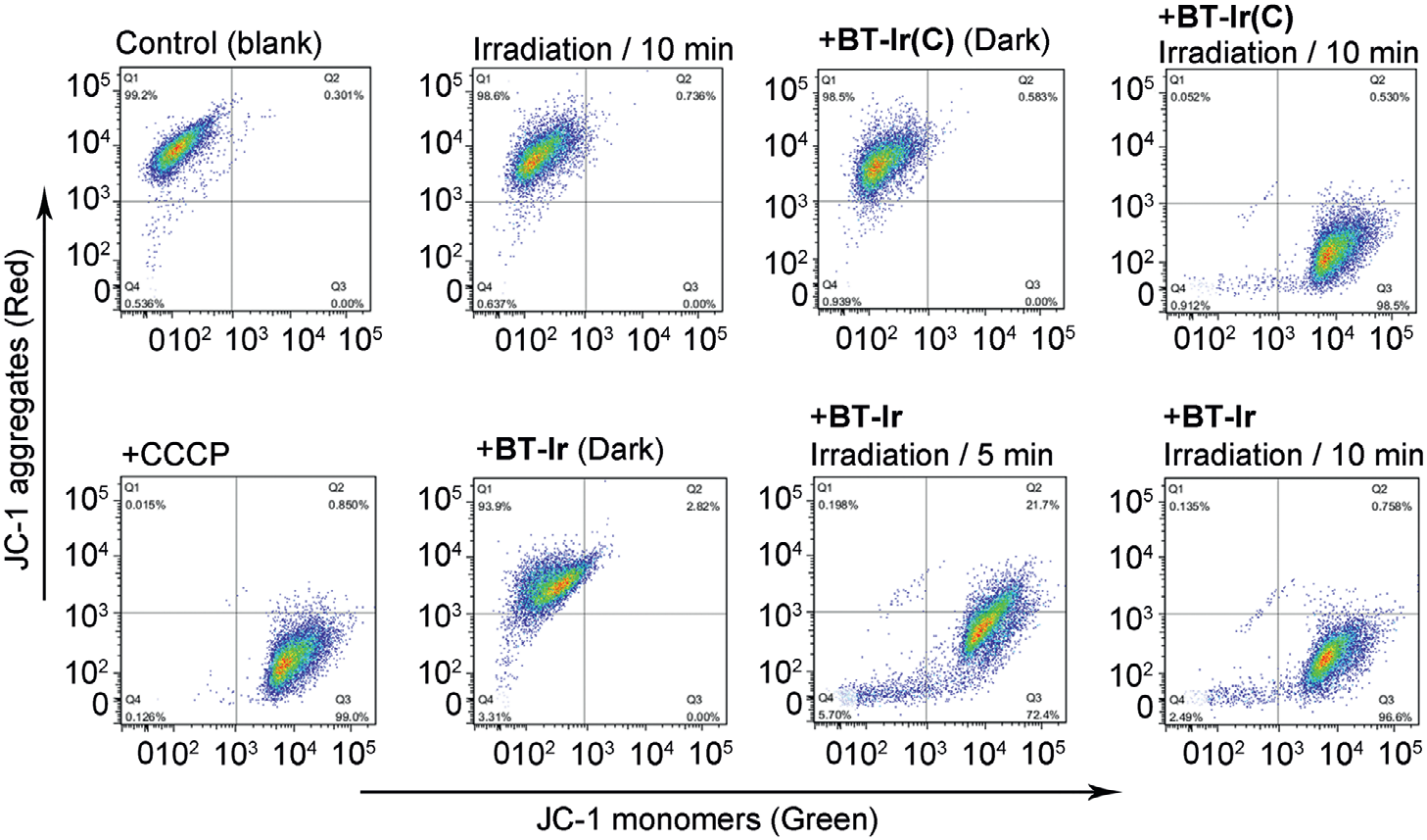
Effects of BT‐Ir and BT‐Ir(C) on MMP were analyzed by flow cytometry under dark or different irradiation time conditions. A549 cells were treated for 30 min, then stained with JC‐1. The commercial drug CCCP, which can induce MMP decrease, was used as a positive control. *λ*_ex_ = 488 nm, *λ*_em_ = 530 nm (green), and 585 nm (red).

The concentration of protons and other ions is distributed asymmetrically on both sides of the mitochondrial inner membrane, and the mitochondria present a negative potential difference, forming MMP.^[^ ^59^ ^]^ The effects of the BT‐Ir and BT‐Ir(C) on the MMP were analyzed by flow cytometry under dark or different irradiation time conditions. A549 cells were first stained with JC‐1 (a membrane‐permeant dye for MMP), BT‐Ir(C) or BT‐Ir, and then irradiated with light for a different time (425 nm LED light 40 mW cm^–2^); cells treated with carbonyl cyanide 3‐chlorophenylhydrazone(CCCP, an agent for decreased MMP) were used as the positive control. As shown in **Figure** **4**, no apparent changes in the MMP were monitored by flow cytometry after incubation of the cells with BT‐Ir(C) or BT‐Ir under dark conductions. However, after light irradiation, the A549 cells stained with the JC‐1 gradually showed green fluorescence, a phenomenon similar to that of the cells induced by CCCP, indicating that both the BT‐Ir(C) and BT‐Ir can induce a decrease in the MMP under irradiation. To further confirm the ROS generation of the BT‐Ir in living cells, 2′,7′‐dichlorofluorescin diacetate (DCFH‐DA) was used as the indicator, which could be rapidly oxidized by the ROS to yield a dichlorofluorescein (DCF) with green fluorescence. As shown in Figure S14, Supporting Information, upon light irradiation, the fluorescence intensity is increased about 6.7‐fold in the presence of the BT‐Ir, which indicates that BT‐Ir could efficiently produce ROS in living cells under light conditions.

**Figure 5:**
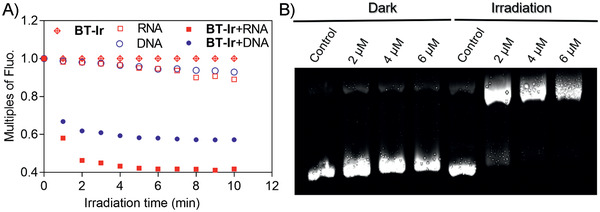
A) The relative fluorescence peak changes of BT‐Ir, DNA/RNA, and BT‐Ir binding with DNA or RNA in 10 mm Tris‐HCl buffer (pH 7.4, 100 mm K^+^) under different irradiation time. B) Electrophoresis experiments of DNA photo‐cleavage of pBR322 plasmid DNA (0.05 µg µL^−1^) treated with different concentrations of BT‐Ir under dark or light irradiation conditions (425 nm LED light 40 mW cm^–2^, 10 min) in 10 mm Tris‐HCl buffer (pH 7.4, 100 mm K^+^).

Once the BT‐Ir molecules are out of the mitochondrial environment, they gradually accumulate in the nucleus due to the hydrogen bonding and electrostatic interaction between DNA/RNA and the positive charge of BT‐Ir.^[^ ^60^, ^61^, ^62^ ^]^ To verify that the BT‐Ir could damage nucleic acids, a series of experiments were carried out. First, stable binding between the BT‐Ir and nucleic acids was observed under dark conditions (Figure S15, Supporting Information). Then, the solution of the BT‐Ir‐nucleic acid complex was exposed under visible light irradiation (425 nm LED, 40 mW cm^–2^, 10 min). As displayed in **Figure** **5**A, the fluorescence of the BT‐Ir decreased significantly in the presence of DNA/RNA upon light irradiation, which indicated serious damage of the DNA/RNA. Further, DNA photocleavage experiments (Figure 5B) showed that upon incubation of pBR322 plasmid DNA with the BT‐Ir under dark conductions, a hindrance of gel mobility of supercoiled DNA was observed. Upon light irradiation, the BT‐Ir induced significant cleavage of the supercoiled configuration, and an increase in the intensity of a nicked band was observed, indicating that the BT‐Ir had a potent DNA cleavage activity under irradiation.

**Figure 6:**
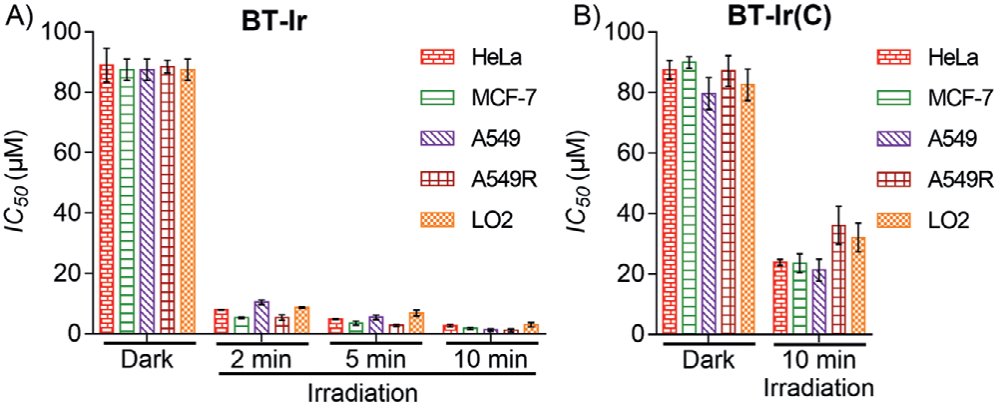
IC_50_ values of tested A) BT‐Ir and B) BT‐Ir(C) towards different cell lines. (IC_50_ values are compounds concentrations necessary for 50% inhibition of cell viability, 425 nm LED light 40 mW cm^–2^. Data are presented as means ± standard deviations, and cell viability is assessed after 24 h of incubation.)

After proving that the BT‐Ir can achieve mitochondria‐to‐nucleus translocation, the cascade double‐killing of cancer cells effect was verified by testing the half‐maximal inhibitory concentration (IC_50_). As shown in **Figure** **6**A, the high IC_50_ values (close to 100 µm) of the BT‐Ir indicate its low dark cytotoxicity towards different living cell lines. Upon light irradiation, the IC_50_ values of the BT‐Ir decreased significantly for all the cell lines tested, proving that the BT‐Ir exhibited high anticancer efficiency in the presence of light irradiation. The results are in sharp contrast with the lower phototoxicity brought by the BT‐Ir(C) (Figure 6B), which has a similar ROS generation ability but cannot be induced into the nucleus under irradiation stimulation. Compared with the BT‐Ir(C), the BT‐Ir shows the light‐driven cascade mitochondria‐to‐nucleoli translocation and enables the use of PSs more effectively in one cell to damage mitochondria and nucleoli, which could reduce dosage and improve photosensitizer efficiency.

**Figure 7:**
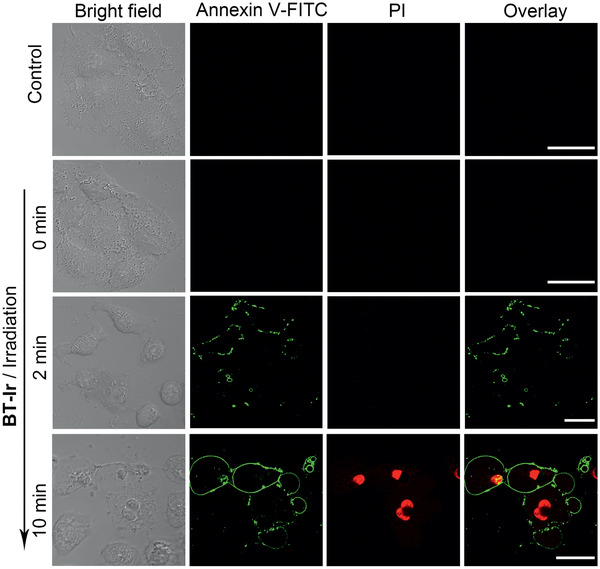
BT‐Ir induced apoptosis in A549 cells. Detection of apoptosis by confocal microscopy after cells were treated with BT‐Ir (10 µm) under different irradiation times with a 425 nm LED light (40 mW cm^–2^). After being stimulated by irradiation, these A549 cells were further incubated for 2 h and then stained with annexin V‐FITC (20.0 µg mL^−1^) and PI (5.0 µg mL^−1^) for 15 min. Scale bar: 20 µm.

Next, an annexin‐FITC (apoptosis staining kit) was used to monitor PDT‐induced cell death process. As shown in **Figure** **7**, before light irradiation, no green fluorescence of annexin V‐FITC on the cell membrane was observed. Upon light exposure, fluorescence from the annexin V‐FITC on the cell membrane gradually enhanced with the increased light exposure time. After 10 min of irradiation, due to increased cell membrane permeability, PI crossed the membrane and entered the nucleus, which means that the cells gradually died. All the results indicate that the death pathway of the cells treated with the BT‐Ir under light irradiation is apoptosis.^[^ ^63^ ^]^

## 3 Conclusion

An iridium complex integrated with a positively charged benzothiazolium unit was developed to target mitochondria, which upon light illumination can achieve the mitochondria‐to‐nucleus cascade cell ablation through dual photosensitization. The BT‐Ir localized in the mitochondria presented low dark toxicity; however, after light induced ROS production, the BT‐Ir can damage the mitochondria and the molecules were subsequently transferred to the nucleus, further damaging the nucleic acids. As compared to the control molecule of BT‐Ir(C), the charge on the benzothiazolium moiety directly contributes to the cascade cell ablation strategy, which improves the anticancer efficiency of the BT‐Ir. This molecular design provides alternative opportunities for achieving efficient photodynamic cell ablation.

## Conflict of Interest

The authors declare no conflict of interest.

## Supporting information

Supporting Information

Supplemental Video 1
