# Supplementary material for: Light‐Driven Cascade Mitochondria‐to‐Nucleus Photosensitization in Cancer Cell Ablation
Source: Adv Sci (Weinh). 2021 Feb 8;8(8):2004379. doi: 10.1002/advs.202004379 (PMC8061408; doi:10.1002/advs.202004379)
Supplement: Supplementary file 1 — Supporting Information [file ADVS-8-2004379-s001.pdf]

© 2021 Wiley-VCH GmbH

ADVANCED SCIENCE
Open Access

Supporting Information

for *Adv. Sci.*, DOI: 10.1002/advs.202004379

Light-driven Cascade Mitochondria-to-nucleus Photosensitization in Cancer Cell Ablation

Kang-Nan Wang, Liu-Yi Liu, Guobin Qi, Xi-Juan Chao, Wen Ma, Zhiqiang Yu, Qi-Ling Pan, Zong-Wan Mao, Bin Liu

Supporting Information

Light-driven cascade mitochondria-to-nucleus photosensitization in cancer cell ablation

Kang-Nan Wang # [a, b], Liu-Yi Liu # [c], Guobin Qi [b], Xi-Juan Chao [c], Wen Ma [e], Zhiqiang Yu*[a,e], Qi-Ling Pan [a], Zong-Wan Mao* [c] and Bin Liu* [b, d]

Table of Contents

Materials and instruments 4
Scheme S1. Synthetic routes of the compounds. 5
Absorption and fluorescence spectra. 7
ROS detection in solution. 7
Hydroxyl radical ($OH^{\bullet}$) detection and singlet oxygen detection. 8
DNA/RNA titration experiments. 8
Molecular docking. 8
Cell lines and culture conditions. 8
Confocal laser scanning microscopy. 9
Cytotoxicity assay. 9
Mito Tracker Deep Red (MTDR) staining. 9
SYTO 59 Red (Syto-R) staining. 10
Analysis of MMP. 10
Intracellular ROS detection. 10
DNA photo-cleavage experiments. 10
Annexin V-FITC/PI staining assay. 11
Statistical analysis. 11
Figure S1. 1H NMR spectrum of **BT-Ir** in DMSO-d6. 12
Figure S2. 13C NMR spectrum of **BT-Ir** in DMSO-d6. 13
Figure S3. ESI-MS spectrum of **BT-Ir** in MeOH. 13
Figure S4. 1H NMR spectrum of **BT-Ir(C)** in DMSO-d6. 14
Figure S5. 13C NMR spectrum of **BT-Ir(C)** in DMSO-d6. 14
Figure S6. ESI-MS spectrum of **BT-Ir(C)** in MeOH. 15
Figure S7. UV-vis absorption spectra of **BT-Ir** recorded in PBS (pH=7.4). 15
Figure S8. Emission spectra of **BT-Ir** recorded in PBS (pH=7.4). 15
Figure S9. The fluorescence changes of **BT-Ir** with increasing concentrations of BSA. 16

Figure S10. The fluorescence changes of **BT-Ir(C)** with increasing concentrations of DNA, RNA, and BSA 16

Figure S11. The ROS generation efficiency of **BT-Ir** in buffer (pH =6.8) 17

Figure S12. Confocal imaging of A549 cells stained with compound **1** and Syto-R. 17

Figure S13. Confocal images of A549 cells stained with **BT-Ir(C)** and MTDR. 18

Figure S14. Intracellular ROS generation efficiency of **BT-Ir** in A549 cells 18

Figure S15. The fluorescence changes of **BT-Ir** binding with DNA/RNA under dark conditions. 19

Table S1. The representative docked free energies of the docking models between **BT-Ir** and DNA/RNA 20

References 21

Materials and instruments

All reagents and solvents (analytical grade) were used as received from commercial sources unless otherwise indicated. Solvents were purified by standard procedures. 3-(4,5-Dimethylthiazol-2-yl)-2,5-diphenyltetrazolium bromide (MTT, Sigma Aldrich), DMSO (Sigma Aldrich), PI (propidium iodide, Sigma Aldrich). 2′,7′-Dichlorofluorescin diacetate (DCFH - DA), carbonyl cyanide 3-chlorophenylhydrazone (CCCP), and Annexin V-FITC apoptosis detection kit were purchased from Sigma Aldrich. 2-Methylbenzothiazole, ammonium hexafluorophosphate, 2-phenylpyridine, iridium (III) chloride hydrate, 1,10-phenanthroline-5,6-dione, 1,4-phthalaldehyde were purchased from Energy Chemical. Piperidine was were purchased from China National Pharmaceutical Group. 9,10-anthracenediyl-bis(methylene) dimalonic acid (ABDA), Singlet Oxygen Sensor Green (SOSG), and 3’-(4-hydroxyphenyl) fluorescein (Molecular Probes Invitrogen, HPF) were obtained from Sigma-Aldrich. The tested compounds were dissolved in DMSO before the experiments, and the concentration of DMSO was 1% (v/v). ^{1}H NMR and ^{13}C NMR spectra were recorded on a Mercury Plus 400 or 600 spectrometer. Shifts are referenced relative to the internal solvent signals. ESI-MS were recorded on a Thermo Finnigan LCQ DECA XP spectrometer. UV–vis spectra were recorded on a Varian Cary 300 spectrophotometer. The quoted m/z values represent the major peaks in the isotopic distribution. Fluorescence microscopy of cells was performed in Carl Zeiss LSM 710. For MTT assays, the absorbance was quantified using an Infinite M200 microplate reader.

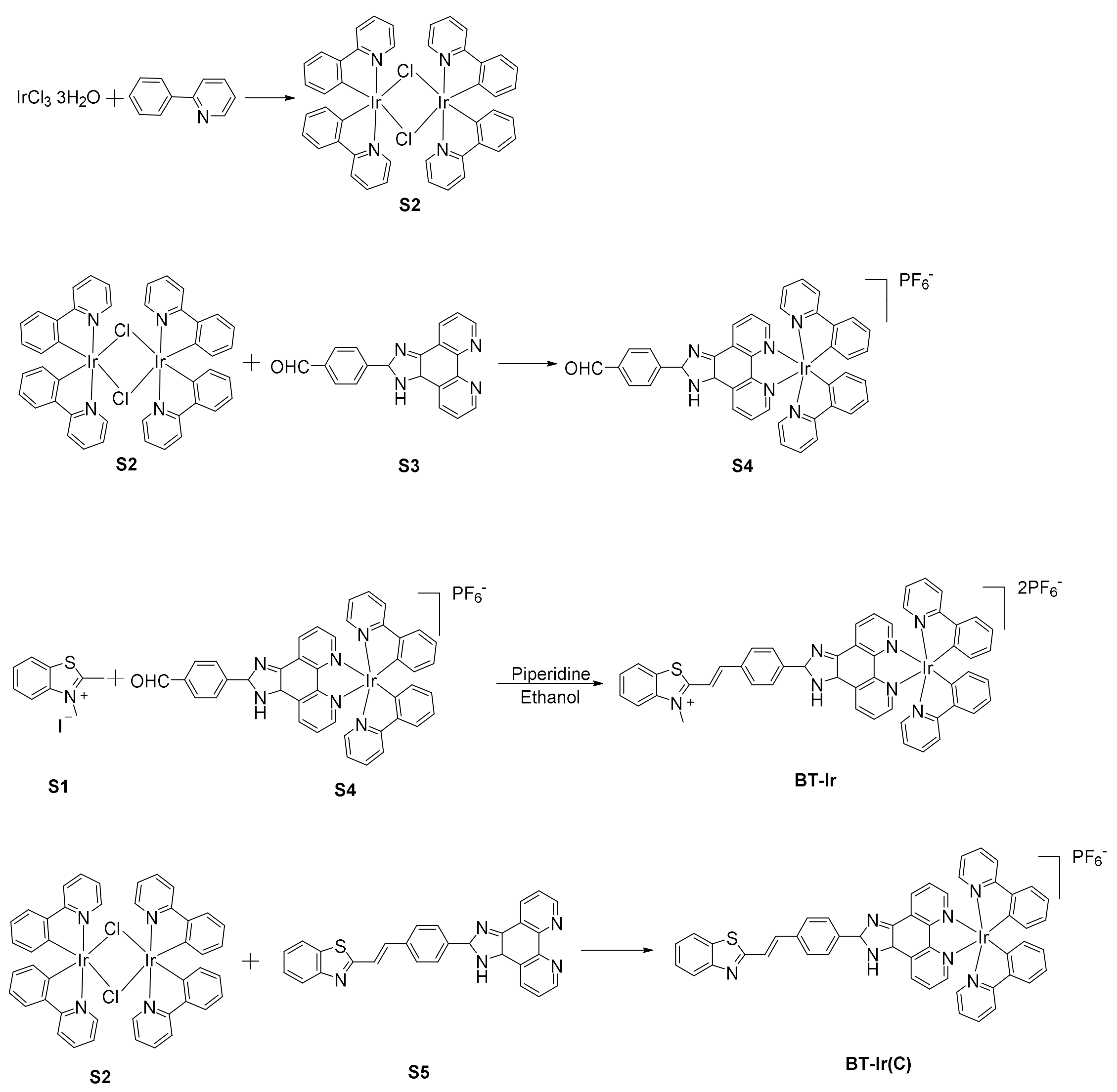

Scheme S1. Synthetic routes of the compounds.

The compound 2,3-dimethylbenzo[d]thiazol-3-ium iodide (**S1**) was synthesized according to the literature method.[1]

The cyclometal iridium (III) chloro-bridged dimer (**$Ir_2(ppy)_4Cl_2$**) (**S2**) and the ligand (4-(2,11b-dihydro-1*H*-imidazo[4,5-*f*][1,10]phenanthrolin-2-yl)benzaldehyde) (**S3**) were prepared according to literature method. [2,3]

Synthesis of S4 {[Ir(ppy)$_2$(S3)](PF$_6$)}. A mixture of **S2** (0.5 mmol, 1 equiv.) and **S3** (1.0 mmol, 2 equiv.) in CH_2Cl_2/CH_3OH was heated to reflux under nitrogen in the dark. After 4 h, the solution was cooled to room temperature, and then a 6-fold excess of NH_4PF_6 was added under stirring, and the mixture was stirred for another 1 h. The

mixture was filtered and evaporated to dryness under reduced pressure. The obtained solid was dissolved in CH_2Cl_2 and purified by column chromatography on silica gel eluted with CH_2Cl_2/acetone. Compound **S4** was obtained as orange solid (1.85g, yield 95%).

Synthesis of BT-Ir {[Ir(ppy)$_2$(S3-BT)](PF$_6$)$_2$}. The ligand **S3-BT** is 2-(4-(2,11b-dihydro-1*H*-imidazo[4,5-*f*][1,10]phenanthrolin-2-yl)styryl)-3-methylbenzo[*d*]thiazol-3-ium. A mixture of **S1** (1 equiv., 0.5 mmol), **S4** (1.1 equiv., 0.55 mmol) and piperidine (50 μL) in ethanol (20 mL) was heated to reflux for 12h. After reaction, the solution was cooled to room temperature; and a 6-fold excess of NH_4PF_6 was added under stirring. The mixture was stirred for another 1 h. Then the mixture was filtered, and the precipitation was washed by ethanol to yield the product. The solid product obtained was further purified by column chromatography on silica gel eluted with CH_2Cl_2 / acetone. Compound **BT-Ir** was obtained as red solid (436 mg, yield 70%). ESI-MS (CH_3OH): m/z calcd for [**BT-Ir**-2PF$_6$]$^{2+}$, 485.6185; found: 485.1572. ^{1}H NMR (600 MHz, DMSO) δ 14.80 (s, 1H), 9.26 (d, *J* = 8.1 Hz, 2H), 8.53 (d, *J* = 8.4 Hz, 2H), 8.49 (d, *J* = 8.1 Hz, 1H), 8.36 – 8.34 (t, 2H), 8.32 (d, *J* = 6.3 Hz, 1H), 8.31 – 8.27 (m, 3H), 8.22 (d, *J* = 15.9 Hz, 2H), 8.20 (s, 1H), 8.14 (dd, *J* = 8.1, 5.1 Hz, 2H), 7.98 (d, *J* = 7.7 Hz, 2H), 7.92 (d, *J* = 9.3 Hz, 1H), 7.91 – 7.88 (m, 2H), 7.83 (t, *J* = 7.6 Hz, 1H), 7.54 (d, *J* = 5.8 Hz, 2H), 7.08 (dd, *J* = 11.3, 4.0 Hz, 2H), 7.01 (dd, *J* = 10.3, 4.1 Hz, 2H), 6.98 (td, *J* = 7.5, 0.8 Hz, 2H), 6.31 (d, *J* = 7.4 Hz, 2H), 4.44 (s, 3H). ^{13}C NMR (126 MHz, DMSO) δ 172.16 (s), 167.37 (s), 152.04 (s), 150.75 (s), 149.65 (s), 149.25 (s), 149.09 (s), 147.55 (s), 144.90 (d, *J* = 15.4 Hz), 144.49 (s), 142.59 (s), 139.20 (s), 137.55 (s), 136.10 (s), 133.29 – 133.06 (m), 133.06 – 132.15 (m), 131.68 (s), 130.88 (d, *J* = 29.9 Hz), 130.56 – 130.50 (m), 129.55 (d, *J* = 116.5 Hz), 128.53 (d, *J* = 15.4 Hz), 127.73 (d, *J* = 40.5 Hz), 126.66 (s), 125.57 (s), 124.83 (s), 124.32 (s), 122.87 (s), 122.35 (s), 120.48 (s), 117.50 (s), 115.56 (s), 37.08 (s).

Synthesis of BT-Ir(C) {[Ir(ppy)$_2$(S5)](PF$_6$)}. The ligand **S5** ((E)-2-(4-(1H-imidazo[4,5-f][1,10]phenanthrolin-2-yl)styryl)benzo[d]thiazole) was

synthesized according to literature method.[4] A mixture of **S2** (0.5 mmol, 1 equiv.) and **S5** (1.0 mmol, 2 equiv.) in CH_2Cl_2/CH_3OH (2:1, v/v) were heated to reflux under nitrogen in the dark. After 4 h, the solution was cooled to room temperature, and then a 6-fold excess of NH_4PF_6 was added under stirring. After that, the mixture was stirred for another 1 h. The mixture was filtered, and evaporated to dryness under reduced pressure. The solid obtained was dissolved in CH_2Cl_2 and purified by column chromatography on silica gel eluted with CH_2Cl_2/acetone. Compound **BT-Ir(C)** was obtained as yellow solid (805 mg, yield 73%). ESI-MS (CH_3OH): m/z calcd for [**BT-Ir(C)**-PF_6]$^+$, 956.2147; found: 956.2543. ^{1}H NMR (400 MHz, DMSO) δ 9.22 (d, *J* = 8.0 Hz, 2H), 8.40 (d, *J* = 8.4 Hz, 2H), 8.28 (d, *J* = 8.3 Hz, 2H), 8.16 (d, *J* = 5.0 Hz, 2H), 8.12 – 7.94 (m, 8H), 7.89 (t, *J* = 7.9 Hz, 2H), 7.75 (d, *J* = 1.7 Hz, 2H), 7.52 (dd, *J* = 12.9, 6.1 Hz, 3H), 7.43 (t, *J* = 7.6 Hz, 1H), 7.02 (dd, *J* = 25.3, 17.0, 7.7 Hz, 6H), 6.31 (d, *J* = 7.5 Hz, 2H). ^{13}C NMR (101 MHz, DMSO) δ 167.37 (s), 150.88 (s), 149.64 (s), 148.81 (s), 144.58 (d, *J* = 14.7 Hz), 139.17 (s), 136.89 (s), 132.80 (s), 131.69 (s), 130.74 (s), 128.89 (s), 127.47 (s), 127.10 (s), 126.08 (s), 125.55 (s), 124.32 (s), 123.46 (s), 122.78 (d, *J* = 13.2 Hz), 120.45 (s), 40.61 (s), 40.40 (s), 40.19 (s), 39.98 (s), 39.77 (s), 39.56 (s), 39.36 (s).

Absorption and fluorescence spectra.

The compounds were dissolved in DMSO to make a 20 mM stock solution. The fluorescence spectra were collected at the range of 410-850 nm upon excitation at 405 nm. For DNA/RNA/BSA titration experiments of **BT-Ir** and **BT-Ir(C)**, small aliquots of DNA/RNA/BSA stock solutions were successively added into the **BT-Ir** or **BT-Ir(C)** solution (10 μM) in 10 mM Tris-HCl buffer (pH 7.4, 100 mM K^+).

ROS detection in solution.

ABDA was used as a ROS indicator to detect the ROS generation in the aerated disodium hydrogen phosphate/citric acid buffer solutions. The ABDA stock solution was mixed with the **[Ru(bpy)$_3$]Cl$_2$**, **BT-Ir(C)**, and **BT-Ir** (10 μM) exposed to light irradiation at a power of 425 nm LED light 40 mW/cm^2 for different times. The

decomposition of ABDA was monitored by the absorbance decrease at 378 nm.

Hydroxyl radical ($OH^{•}$) detection and Singlet oxygen detection.

The hydroxyl radical ($OH^{•}$) and singlet oxygen were detected according to the literature method.[5]

DNA/RNA titration experiments.

BT-Ir or **BT-Ir(C)** solutions were initially prepared in the solvents of DMSO with a concentration of 20 mM. The stock solution of DNA, RNA, and BSA were prepared in the concentration of 1 mg/mL in 10 mM Tris-HCl buffer (pH 7.4, 100 mM K^{+}). **BT-Ir /BT-Ir(C)** (10 μM) and different concentrations of DNA, RNA, and BSA were added into the Tris-HCl buffer (pH 7.4, 100 mM K^{+}). Then the fluorescence spectra were also measured with the spectrofluorometer with the excitation wavelength of 405 nm.

Molecular docking.

DNA/RNA preparation: the structure of the DNA/RNA-ligand complex was downloaded from RCSB Protein Data Bank (PDB code: 5ju4 for DNA; RNA: PDB code: 2lwk for RNA). The original ligand and water were removed by PyMOL[6], and the DNA/RNA was prepared by Accelrys Discovery Studio 2.5.5[7] for docking studies. Ligand preparation: **BT-Ir** is optimized using DFT calculations by the Gaussian09 package[8] at B3LYP/6-31g (d, p) level. Using the optimized **BT-Ir** structure, the partial atomic charges were obtained by restrained electrostatic potential (RESP)[9] calculating with the Gaussian 09 package at the level of HF/6-31g*. After that, the docking calculations were conducted by the AutoDock 4.2 suite of programs [10] with a ligand flexible docking approach. The Lamarckian genetic algorithm [11] was chosen as the search protocol using the default parameters except for the number of GA runs (ga_run = 80) and the maximum number of energy evaluations (ga_num_evals = 25,000,000). The docking model, with the 20 lowest docked free energy, was selected for further investigation in this article. The displaying images were rendered with PyMOL.

Cell lines and culture conditions.

A549 cells, MCF-7 cells, A549R cells, LO2 cells, and HeLa cells were obtained from American Type Culture Collection (ATCC). Cells were routinely maintained in DMEM (Dulbecco's modified Eagle's medium, Gibco BRL) or RPMI 1640 (Roswell Park Memorial Institute 1640, Gibco BRL) medium containing 10% FBS (fetal bovine serum, Gibco BRL), 100 μg/mL streptomycin, and 100 U/mL penicillin (Gibco BRL). The cells were cultured in tissue culture flasks in a humidified incubator at 37 °C, in an atmosphere of 5% CO_2 and 95% air. In each experiment, the cells treated with DMSO (1%, v/v) were used as the reference group.

Confocal laser scanning microscopy.

A549 cells in 8-well chambers with 5×10^4 cells per well were prepared according to the above description. Cells were cultured in an incubator overnight and then treated with **BT-Ir** or **BT-Ir(C)** (10 μM) at each well. **BT-Ir** or **BT-Ir(C)** were incubated for 90 min, respectively. After incubation, the cells were subjected to fluorescence microscopy of cells (Carl Zeiss LSM 710).

Cytotoxicity assay.

Cells cultured in 96-well plates were grown to confluence. The compounds were dissolved in DMSO (1%, v/v), and diluted with fresh media immediately. Then the cells were incubated with different concentrations of the tested compounds for 20 h at 37 °C. 20 μL of MTT solution was then added to each well, and the plates were incubated for an additional 4 h. The medium was carefully removed, and DMSO was added (150 μL per well). The plates were incubated for 10 min with shaking. The absorbance at 595 nm was measured using a microplate reader (Infinite M200 Pro, Tecan, Switzerland).

Mito Tracker Deep Red (MTDR) staining.

A549 cells were seeded in 8-well chambers with 5×10^4 cells per well and incubated overnight at 37°C. Then the cells were further incubated with **BT-Ir** or **BT-Ir(C)** (10 μM) and MTDR (500 nM) for 30 min before visualized by confocal microscopy. For **BT-Ir**, λ_{ex}: 405 nm; λ_{em}: 620 ± 20 nm; For **BT-Ir (C)**, λ_{ex}: 405 nm; λ_{em}: 500 ± 20 nm;

For MTDR, λ_{ex}: 633 nm; λ_{em}: 650 ± 10 nm.

SYTO 59 Red (Syto-R) staining.

A549 cells were seeded in 8-well chambers with 5×10^4 cells per well and incubated overnight at 37°C. Then the cells were further incubated with **BT-Ir** (10 μM) and **Syto-R** (3 μM/mL) for 20 min before visualization with confocal microscopy. For **BT-Ir**, λ_{ex}: 405 nm; λ_{em}: 620 ± 20 nm; For **Syto-R**, λ_{ex}: 633 nm; λ_{em}: 650 ± 10 nm.

Analysis of MMP.

For flow cytometry, A549 cells were seeded into 6 well plates and cultured for 48 h. After treated with **BT-Ir**, **BT-Ir(c)**, and CCCP at the indicated concentrations for 90 min, and then irradiated with a 425 nm LED light (40 mW cm^{-2}) for a different time for **BT-Ir** and **BT-Ir(c)**. The cells were harvested and resuspended in a pre-warmed staining buffer containing 5 μg/mL JC-1 (1 mL) and incubated at 37 °C for 20 min. Subsequently, the cells were washed twice with blank buffer and immediately measured by flow cytometry at excitation at 488 nm and dual emission at 530 nm (FITC, Green) and 585 nm (PE, Red). Mean fluorescence intensity (MFI) was analyzed using FlowJo 7.6.1 software (Tree Star, OR, USA). 10, 000 cells were acquired for each sample.

Intracellular ROS detection.

The ROS generation inside the cells upon light irradiation was studied using a cell-permeable indicator DCF-DA. A549 cells in 8-well chambers (Thermo Scientific) were firstly incubation with the **BT-Ir** (10 μM) and DCFH-DA (10 μM) for 30 min, and then the cells were washed with 1× PBS and under different irradiation time with a 425 nm LED light for (40 mW cm^{-2}) for a different time. After irradiation, the cells were washed with 1× PBS and studied by confocal microscope. For DCF detection, the excitation was 488 nm, and the emission filter was 510-550 nm.

DNA photo-cleavage experiments.

Supercoiled pBR322 plasmid DNA (0.05 μg/μL) was incubated with an increasing concentration of **BT-Ir** (2, 4, and 6 μM) in buffer solution (10 mM Tris-HCl, pH = 7.4) at 37 °C for 30 min in the dark. Then the solutions were irradiated with 425 nm LED light (40 mW cm^{-2}, 10 min) or remained in the dark, after which the samples were incubated at 37 °C for another 2 h in the dark. The reactions were then quenched by adding 2 μL of 6 × loading buffer (Takara, Japan). The resulting mixtures were loaded onto a 0.9% agarose gel containing EB. Following the electrophoresis at 120 mV for 2 h in TBE (Tris-borate-EDTA) buffer, the bands were visualized and photographed using UVP Bio-Imaging Systems.

Annexin V-FITC/PI staining assay.

The assay was performed according to the manufacturer's (Sigma Aldrich, USA) protocol. For confocal microscopy analysis, A549 cells were seeded in 8-well chambers with 5×10^4 cells per well for 12 h. After incubation with **BT-Ir** (5 μM) at 37 °C for 30 min, cells were washed with ice-cold PBS three times and irradiated with light for different times. After that, cells were further incubated with 200 μL of annexin-binding buffer supplemented with 5 μL of Annexin V-FITC (20 μg/mL) and 10 μL of PI (5 μg/mL) for 15 min in the dark before confocal microscopy imaging.

Statistical analysis.

All biological experiments were performed at least twice with triplicates in each experiment. Representative results were depicted in this report, and data were presented as means ± standard deviations (SD) with statistical significance.

Supporting Figures and Tables

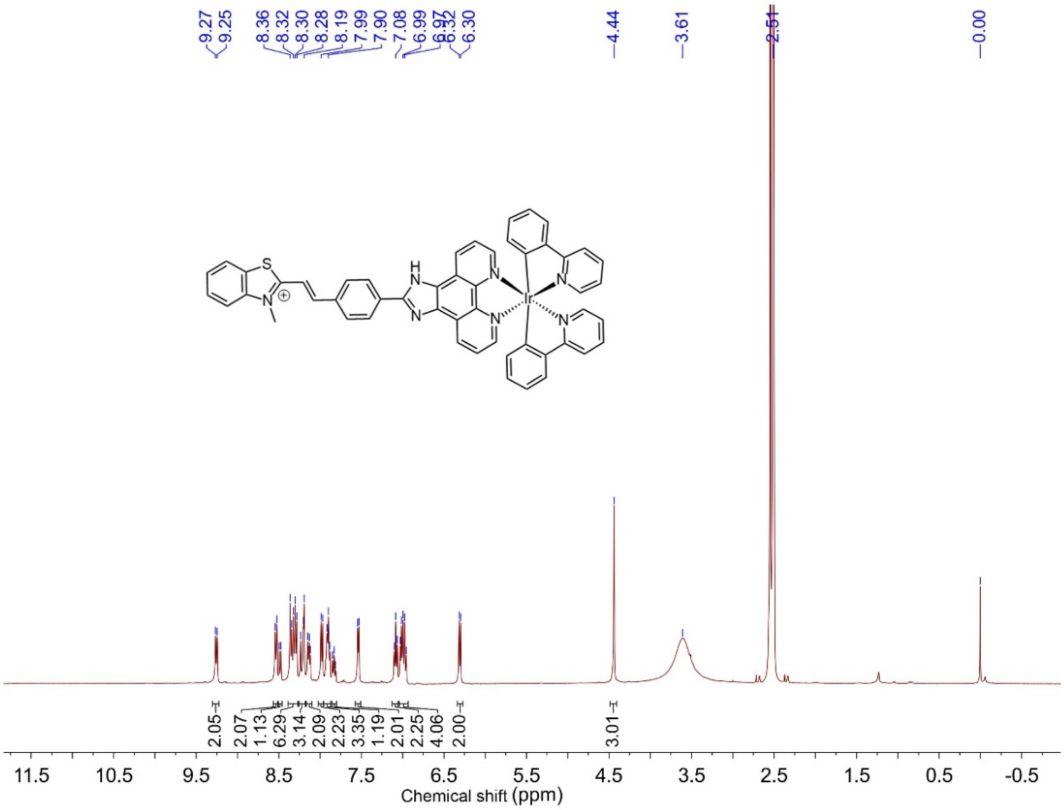

Figure S1. ^{1}H NMR spectrum of **BT-Ir** in DMSO-d_6.

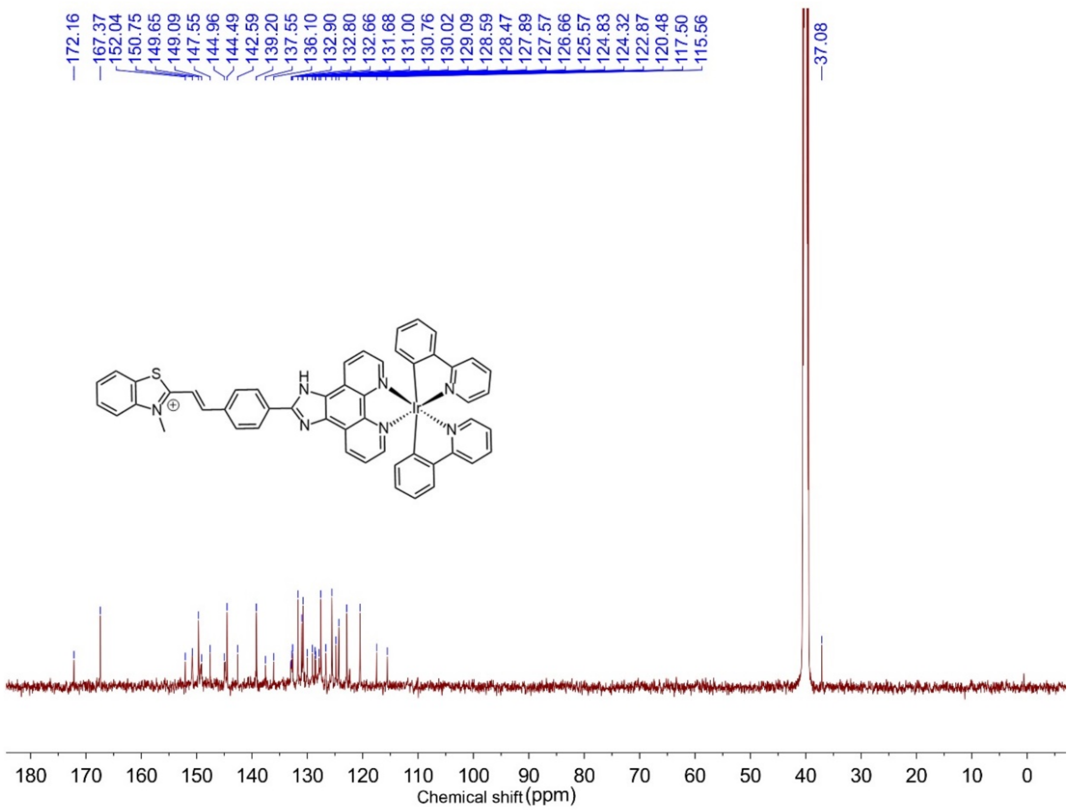

Figure S2. ^{13}C NMR spectrum of **BT-Ir** in DMSO-d_6.

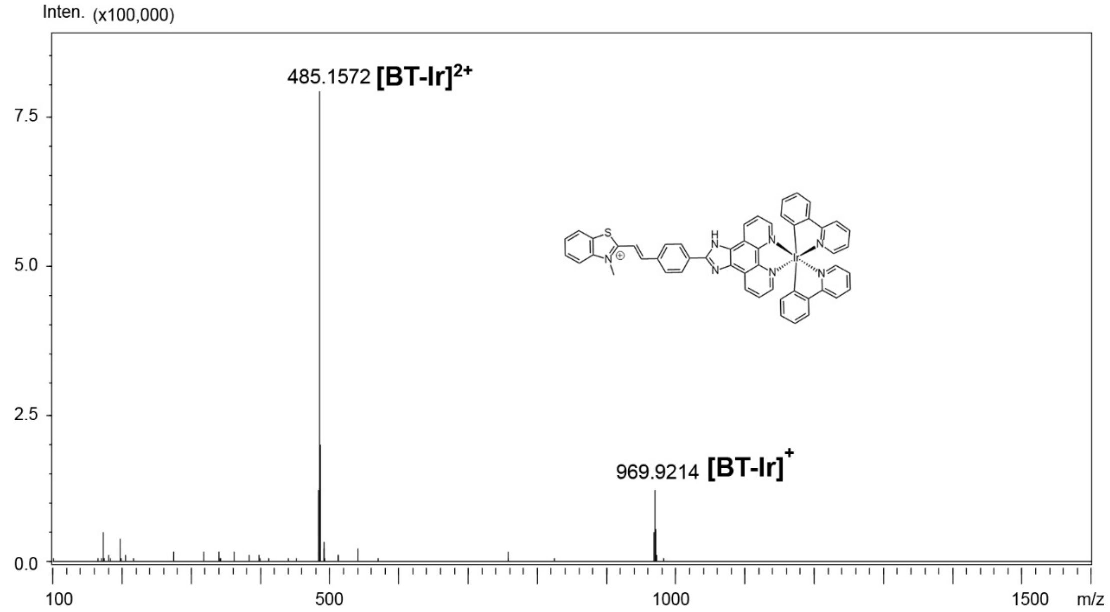

Figure S3. ESI-MS spectrum of **BT-Ir** in MeOH.

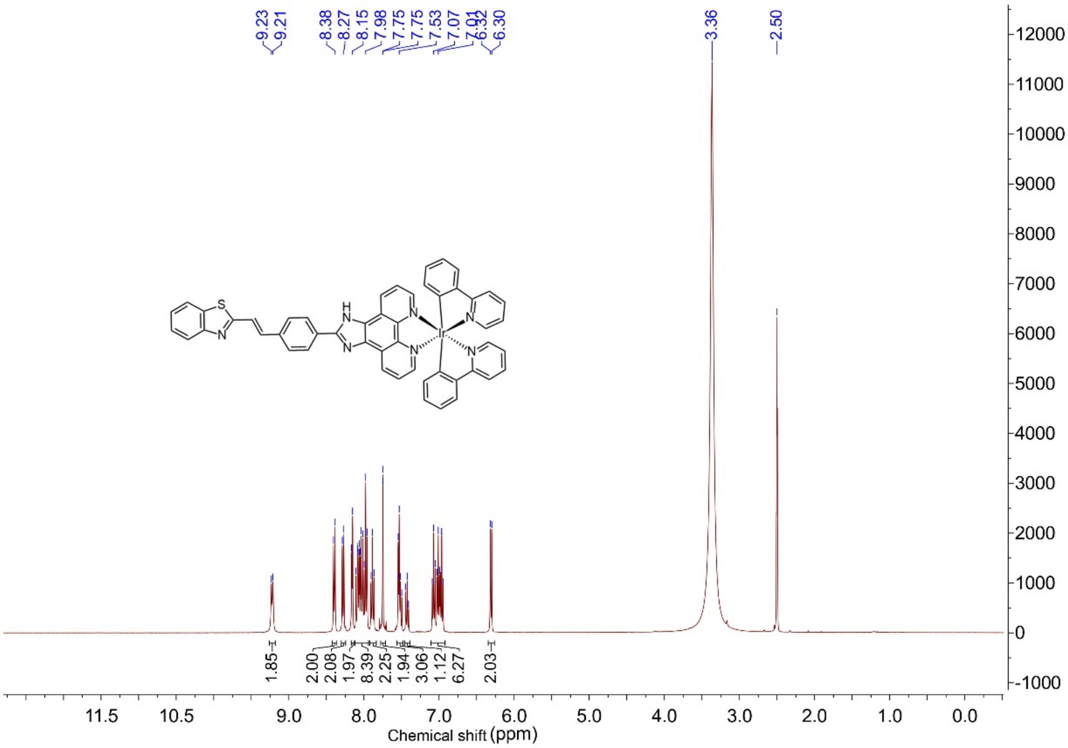

Figure S4. ^{1}H NMR spectrum of **BT-Ir(C)** in DMSO-d_6.

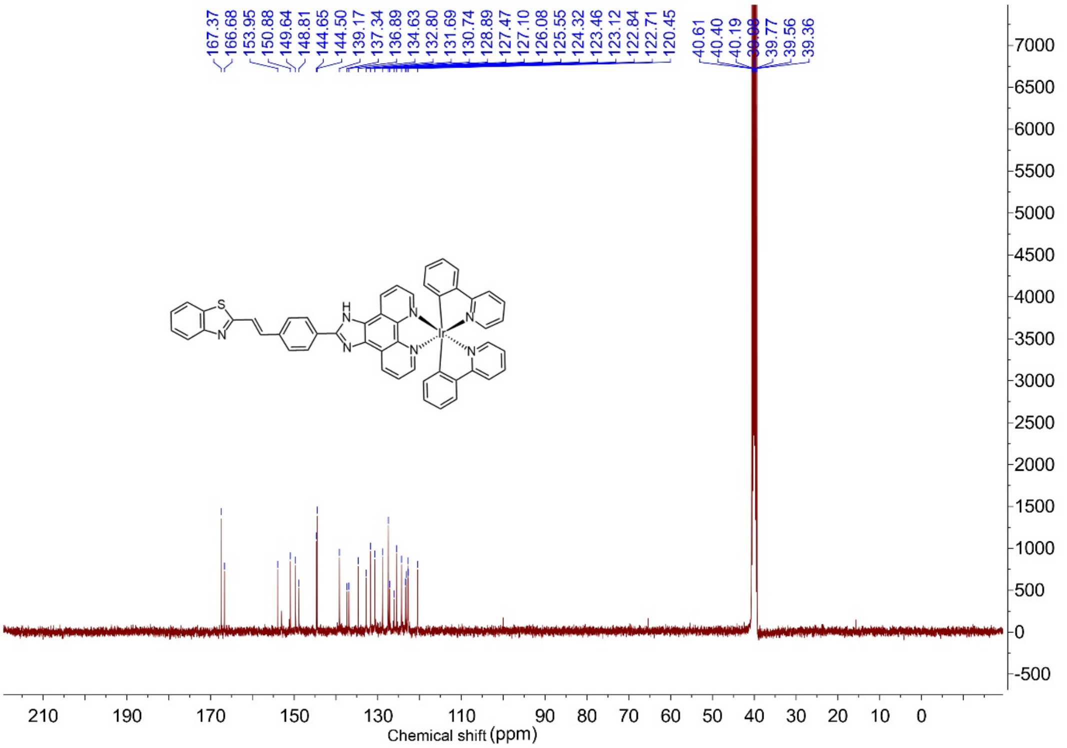

Figure S5. ^{13}C NMR spectrum of **BT-Ir(C)** in DMSO-d_6.

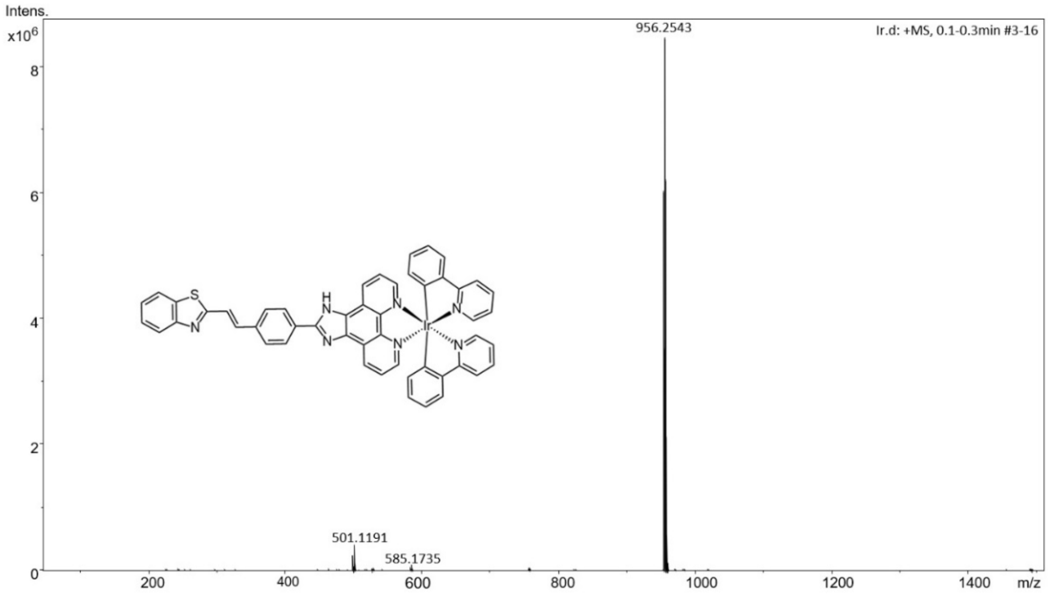

Figure S6. ESI-MS spectrum of **BT-Ir(C)** in MeOH.

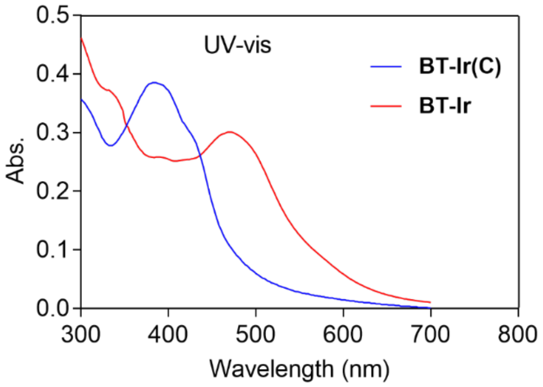

Figure S7. UV/Vis absorption spectra of **BT-Ir(C)** and **BT-Ir** (10 μM) recorded in PBS (pH=7.4).

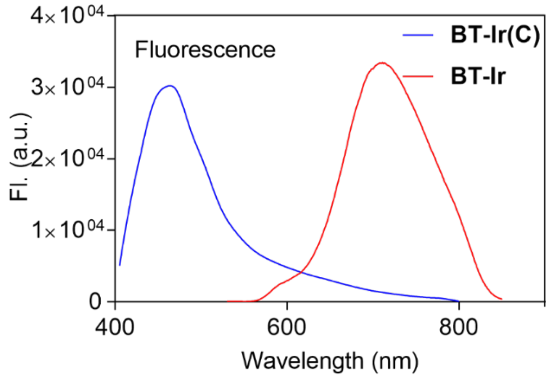

Figure S8. Emission spectra of **BT-Ir(C)** and **BT-Ir** (10 μM) recorded in PBS

(pH=7.4). λ_{ex} = 405 nm.

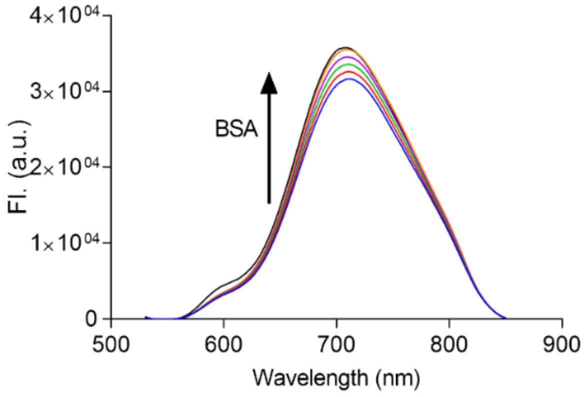

Figure S9. The fluorescence changes of **BT-Ir** (10 μM) in Tris-HCl buffer (pH 7.4, 100 mM K^+) with increasing concentrations of bovine serum albumin (BSA).

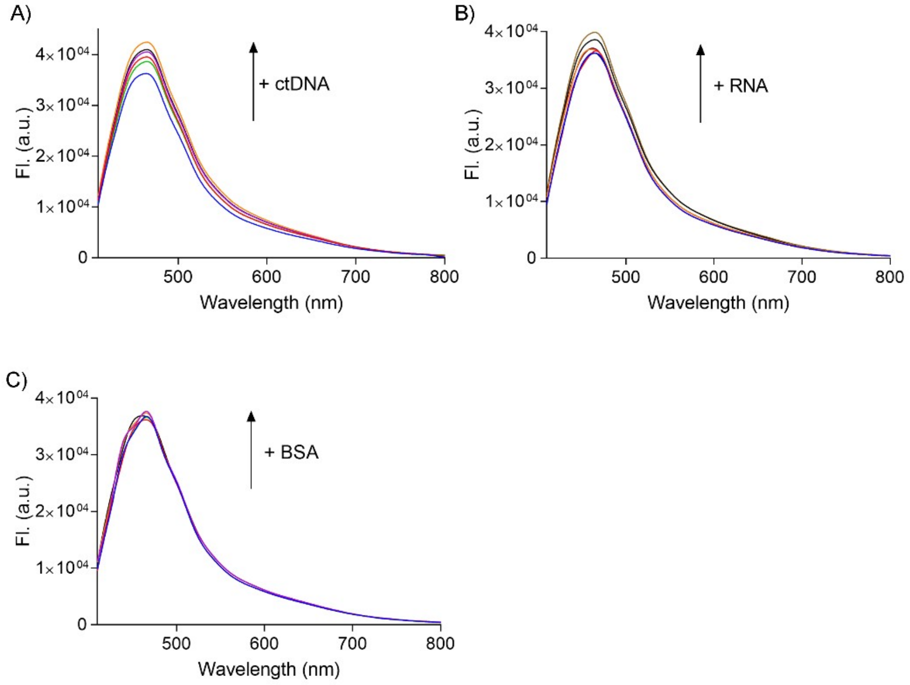

Figure S10. The fluorescence spectra of **BT-Ir(C)** (10 μM) in Tris-HCl buffer (pH 7.4, 100 mM K^+) with increasing concentrations of DNA (A), RNA (B), and BSA (C).

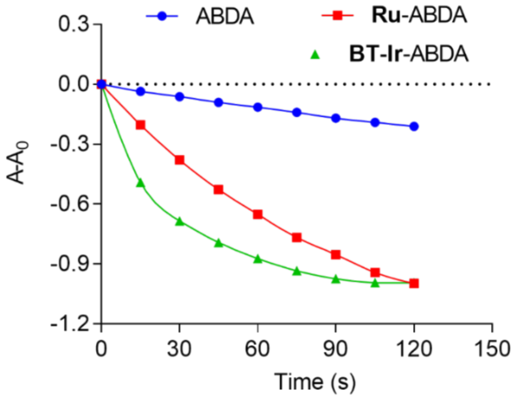

Figure S11. Rate of decay of ABDA sensitized by control, Ru ([Ru(bpy)$_3$]Cl_2), **BT-Ir** in aerated disodium hydrogen phosphate/citric acid buffer solutions as shown by the decrease in the absorption maxima of ABDA for pH =6.8, where A_0 and A are the absorbances of ABDA at 378 nm before and after light irradiation.

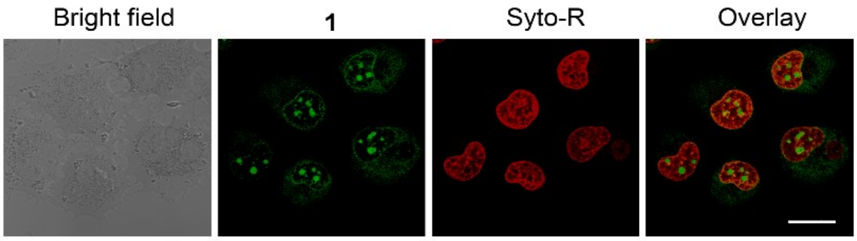

Figure S12. Confocal imaging of A549 cells stained with compound **1** (10 µM) and Syto-R (500 nM). For compound **1**, λ_{ex}: 488 nm; λ_{em}: 530 ± 20 nm. For Syto-R, λ_{ex}: 633 nm, λ_{em}: 650 ± 10 nm. Scale bar: 20 µm.

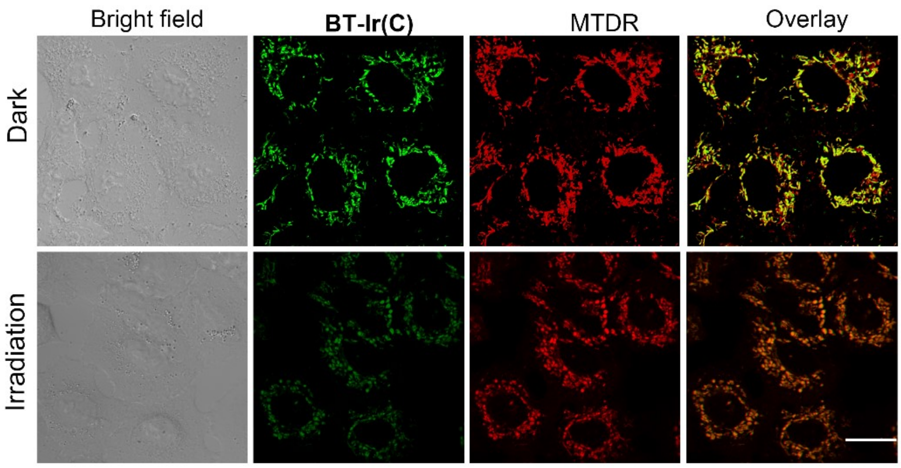

Figure S13. Confocal images of A549 cells stained with **BT-Ir(C)** (10 μM) and MTDR (500 nM) for 30 min, before and after 405 nm light irradiation for 5 min. Scale bar: 20 μm. For **BT-Ir(C)**, λ_{ex}: 405 nm; λ_{em}: 500 ± 20 nm; For MTDR, λ_{ex}: 633 nm; λ_{em}: 650 ± 10 nm.

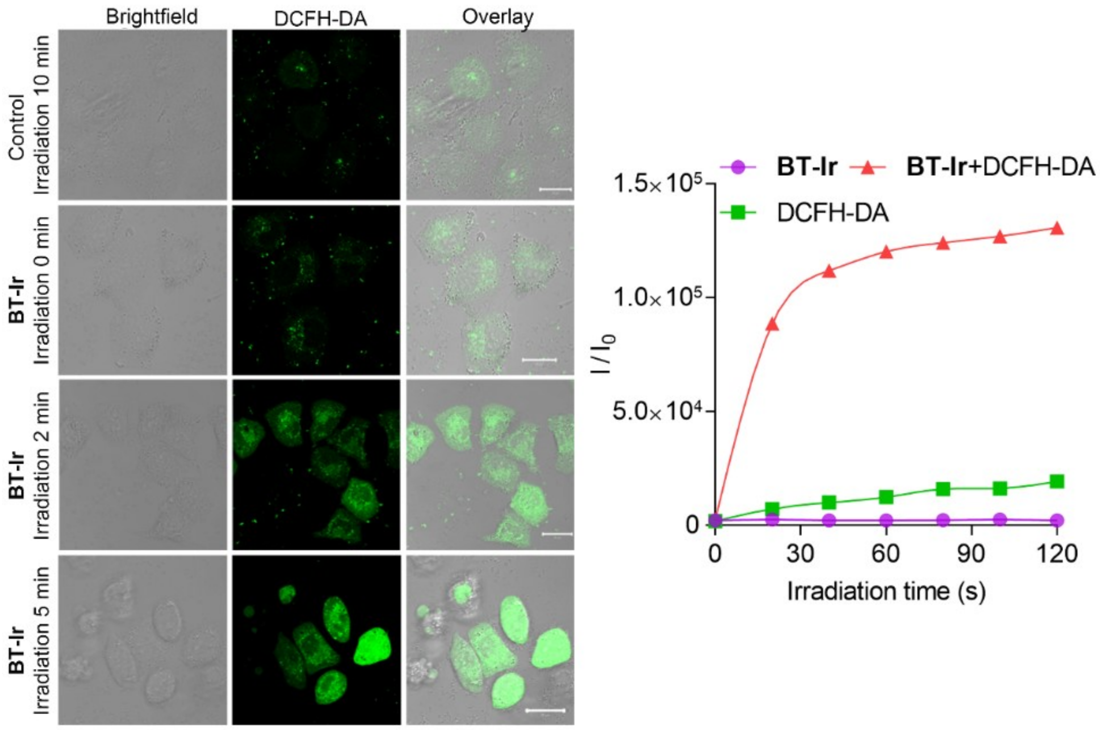

Figure S14. Intracellular ROS production using DCFH-DA (10 μM) in A549 cells after a 30 min treatment with **BT-Ir** (10 μM) at the indicated concentrations under different irradiation time with a 425 nm LED light (40 mW cm^{-2}) for different time. For DCF

detection, the excitation was 488 nm, and the emission filter was 510 − 550 nm. Scale bar: 20 μm.

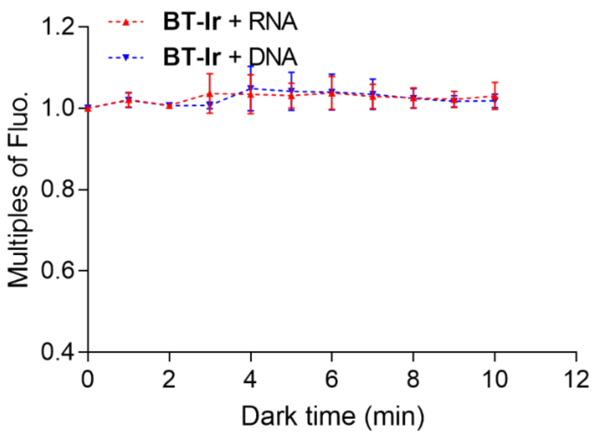

Figure S15. The fluorescence peak changes of **BT-Ir** (10 μM) binding with DNA or RNA in PBS under dark conditions.

Table S1. The representative docked free energies of the docking models between **BT-Ir** and DNA/RNA, and H-bonds between the polar hydrogen of the phenolic hydroxyl group of **BT-Ir** and the oxygen of the phosphate group of DNA/RNA.

DNA			RNA		
Pose	Docked free energy (kcal/mol)	Distance of H-bonds (Å)	Pose	Docked free energy (kcal/mol)	Distance of H-bonds (Å)
1	-11.60	2.13	1	-9.34	1.87
2	-11.59	2.14	2	-9.34	1.87
3	-11.57	2.10	3	-9.33	1.89
4	-11.57	2.14	4	-9.32	1.88
5	-11.56	2.14	5	-9.22	1.82
6	-11.56	2.14	6	-9.03	1.87
7	-11.55	2.16	7	-8.95	1.79
8	-11.54	2.13	8	-8.85	2.08
9	-11.54	2.16	9	-8.85	2.06
10	-11.54	2.14	10	-8.85	2.06
11	-11.54	2.14	11	-8.84	2.05
12	-11.53	2.13	12	-8.83	2.04
13	-11.53	2.15	13	-8.85	2.10
14	-11.53	2.14	14	-8.77	2.07
15	-11.53	2.16	15	-8.85	2.06
16	-11.53	2.16	16	-8.72	1.93
17	-11.52	2.16	17	-8.72	1.94
18	-11.52	2.17	18	-8.71	1.95
19	-11.52	2.17	19	-8.71	1.95
20	-11.52	2.17	20	-8.71	1.95

References

[1] Perdisatt L, Moqadasi S, O'Neill L, et al. *J. Inorg. Biochem.*, **2018**, 182: 71-82.

[2] J. Dai; Z. Q. Liu; X. Q. Wang; et al. *J. Med. Chem.* **2015**, *58* (9), 3875-3891.

[3] C. Y. Li, M. X. Yu, Y. Sun, Y. Q. Wu, C. H. Huang, F. Y. Li, *J. Am. Chem. Soc.* **2011**, *133*, 11231-11239.

[4] Y.L. Pan, Z.B. Cai, L. Bai, F.F. Ma, S.L Li,, Y.P. Tian, *Tetrahedron*, **2017,** 73: 2886-2893.

[5] M. Li, J. Xia, R. Tian, J. Wang, J. Fan, J. Du, S. Long, X. Song, J. W. Foley, X. Peng, *J. Am. Chem. Soc.* **2018**, 140, 14851−14859

[6] W. L. DeLano, PyMOL(TM) Molecular Graphics System, Version 1.7 (Schrodinger, LLC, **2009**).

[7] Accelrys Discovery Studio, Version 2.5.5 (Accelrys Software Inc., San Diego, CA, **2010**).

[8] M. J. Frisch, et al. Gaussian 09, Revision D.01. (Gaussian Inc., Wallingford CT, **2013**).

[9] C. I. Bayly, P. Cieplak, W. Cornell, P. A. Kollman, *J. Phys. Chem.*, **1993**, 97, 10269-10280.

[10] G. M. Morris, R. Huey, W. Lindstrom, , et al. *J. Comput. Chem.,* **2009**, 30, 2785-2791.

[11] G. M. Morris, D. S. Goodsell, R. S. Halliday, , et al. *J. Comput. Chem.,* **1998**, 19, 1639-1662.
