# Supplementary figures and images for: Light‐Driven Cascade Mitochondria‐to‐Nucleus Photosensitization in Cancer Cell Ablation
Source: Adv Sci (Weinh). 2021 Feb 8;8(8):2004379. doi: 10.1002/advs.202004379 (PMC8061408; doi:10.1002/advs.202004379)

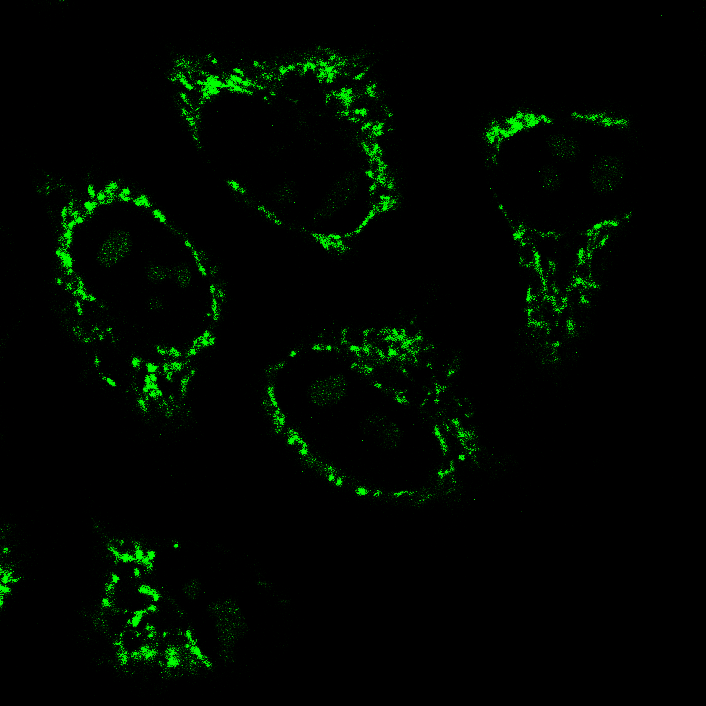

Supplement: Supplementary file 2 — Supplemental Video 1 [file ADVS-8-2004379-s002.gif]
